# Frequency and Factors Associated with Hyperglycaemia First Detected during Pregnancy at Itojo General Hospital, South Western Uganda: A Cross-Sectional Study

**DOI:** 10.1155/2020/4860958

**Published:** 2020-08-13

**Authors:** Frank Kiiza, Daniel Kayibanda, Pidson Tumushabe, Leticia Kyohairwe, Raymond Atwine, Rogers Kajabwangu, Ritah Kiconco

**Affiliations:** ^1^Department of Medical Laboratory Science, Faculty of Medicine, Mbarara University of Science and Technology, Mbarara 1410, Uganda; ^2^Department of Pathology, Faculty of Medicine, Mbarara University of Science and Technology, Mbarara 1410, Uganda; ^3^Department of Obstetrics and Gynaecology, Kampala International University-Teaching Hospital, Ishaka Bushenyi 71, Uganda; ^4^Department of Medical Laboratory Science, School of Allied Health Science, Kampala International University-Western Campus, Ishaka Bushenyi 71, Uganda

## Abstract

**Background:**

Hyperglycemia in pregnancy complicates up to 30% of pregnancies in Africa, and this poses a major risk to both the mother and fetus. Although recommended by the World Health Organization (WHO), universal screening for hyperglycaemia in pregnancy is not routinely done in many of the hospital in low- and middle-income countries. This study sought to determine the frequency and factors associated with hyperglycaemia first detected in pregnancy at a general public hospital in South Western Uganda.

**Methods:**

We conducted this study at Itojo General Hospital (IGH) in Ntungamo District, South Western Uganda. The study followed a cross-sectional design that employed a systematic random sampling technique to identify potential study participants during the months of October to December 2019. Using a pretested questionnaire, data on sociodemographic and medical characteristics were collected on a sample of 307 pregnant women. Blood samples were collected and analyzed for hyperglycemia using random blood sugar and fasting blood sugar test results. Data generated were analyzed with appropriate statistical tests using the statistical package for social sciences (SPSS) version 26 (SPSS Inc., USA) at *P* < 0.05.

**Results:**

The frequency of hyperglycaemia was found to be 15.6% among the study population. Multivariate analysis revealed that the ages of 19-30 years, peasantry, and multiparity with more than 5 live children and second trimester pregnancy were independent risk factors for the observed hyperglycaemia frequency.

**Conclusion:**

Our study reports new epidemiological information about the frequency and risk factors of hyperglycaemia in pregnancy from a selected Ugandan population. Our findings suggest an introduction of hyperglycaemia screening in the routine antenatal care package for proper maternal and neonatal health outcomes.

## 1. Introduction

Hyperglycaemia is a metabolic condition resulting from defects in insulin secretion, insulin action, or both. Chronic hyperglycaemia has been documented as the key clinical characteristic feature in diabetes mellitus and is associated with the long-term damage, dysfunction, and failure of different organs especially the eyes, kidneys, nerves, heart, and blood vessels [1]. Diabetes mellitus can be classified into Type 1, Type 2, and gestational diabetes mellitus. Type 1 diabetes mellitus is a result of cellular-mediated autoimmune destruction of the beta cells of the pancreas, causing an absolute deficiency of insulin secretion. On the other hand, Type 2 diabetes mellitus is as a result of an individual's resistance to insulin with an insulin secretory defect [2]. Gestational diabetes mellitus (GDM) is defined as any degree of glucose intolerance with onset or first recognition during pregnancy. However, this definition applies whether insulin or only diet modification is used for treatment and whether or not the condition persists after pregnancy. Also, it does not exclude the possibility that unrecognized glucose intolerance may predate the pregnancy [3]. The precise mechanisms underlying gestational diabetes remain unknown although the hallmark of the disease was found to be hyperglycaemia due to increased insulin resistance [3, 4].

It is prudent to note that the frequency of hyperglycaemia first detected in pregnancy varies worldwide and among ethnic groups depending upon the population studied and the used diagnostic tests. The worldwide frequency of hyperglycaemia in pregnancy as of 2014 was estimated at 16.9% with majority of contributing cases from low-middle-income countries at 13.4% [5]. Information about hyperglycaemia first detected in pregnancy in Africa is limited. This emphasizes the need to prioritize the importance of investigating the condition from a public health, maternal and child health perspective, particularly in developing countries such as Uganda. A study conducted in Uganda acknowledged the fact that according to World Health statistics, there is no country data available about the specific frequency and factors associated with hyperglycaemia first detected in pregnancy. However, the estimates modelled using data from other countries and specific country characteristics showed the frequency of raised fasting blood glucose among females aged ≥25 years was 6.5% which can be used to uphold the reality that hyperglycemias in pregnancy and its complications do exist and pose a serious health challenge [6]. A high frequency of 31.9% of hyperglycaemia in a population of pregnant women receiving healthcare at St. Francis Nsambya Hospital in Uganda was registered [6]. Another study noted that approximately 7% of all pregnancies are complicated by hyperglycaemia resulting into more than 200,000 cases of Gestational Diabetes Mellitus (GDM) annually [4]. A significant proportion of these women were diagnosed with GDM, and 23.8% of them had hyperglycaemia without any specific risk factor and that the frequency of gestational diabetes affected 3-10% of pregnancies [7].

The risk of complications of hyperglycaemia in pregnancy cannot be exclusive to mothers alone but rather extrapolated to the newborns as well. Researchers investigating the global frequency of hyperglycaemia in pregnancy enlisted the short-term complications of the condition on the infants born to mothers with hyperglycaemia as having an increased risk of foetal macrosomia (also known as large for gestational age), hypoglycaemia, and hyperinsulinemia at birth and risks of shoulder dystocia associated with obstructed labour, and the list goes on. Mothers with hyperglycaemia on the other hand face the increased risk to preeclampsia, gestational hypertension, caesarean section, and hydramnios [5]. According to the World Health Organization report of 2006, about 40-60% of women with gestational diabetes mellitus are associated with demonstrable risk factor. For this reason, many health programs should advocate to screen all pregnant women for hyperglycaemia. Typically, women with gestational diabetes mellitus exhibited no symptoms (another reason for universal screening), but some women might demonstrate increased thirst, increased urination, fatigue, nausea and vomiting, blurred vision, high susceptibility to urinary tract infection, and yeast infections [8]. The risk factors for gestational diabetes mellitus diagnosed as hyperglycaemia were advanced age (≥35 yrs), overweight or obesity, excessive gestational weight gain, excessive central body fat deposition, family history of diabetes, short stature (<1.50 m), excessive foetal growth, polyhydramnios, hypertension or preeclampsia in the current pregnancy, history of recurrent miscarriage, offspring malformation, foetal or neonatal death, macrosomia, and polycystic ovary syndrome [9].

There are quite a number of benefits of knowing the frequency of hyperglycaemia in pregnancy, but what stands out most is the fact that failure to recognize the impact of current statistics on the status of hyperglycaemia in pregnancy could lead to future high frequency of the condition and thus contribute to an increased disease burden on a given health system, and Uganda is no exception. It is worth noting that women with hyperglycaemia are at high risk of hypertension, abortion/miscarriage, and/or a pregnancy resulting in a newborn that is large for gestational age (macrosomia), preterm birth, or neonatal death. Moreover, the researchers further stress that sedentary lifestyle, maternal height, dietary factors, cigarette smoking, and extreme pregnancy weight gain accompanied by high body fat accumulation could put women at risk of being diagnosed with hyperglycaemia in pregnancy [10]. Therefore, early detection of hyperglycaemia in pregnancy could provide relevant information to the attending gynaecologists to properly manage a particular pregnant woman.

This current study was conducted in Itojo General Hospital which is a public health facility and the main hospital in Ntungamo District in South Western Uganda. The facility is the major catchment area for most of the villages in the district, and due to the free health care services, it is the most attended hospital for antenatal care in the region receiving a substantial number of pregnant women on a daily basis. The situation at the facility led us to determine the frequency of hyperglycaemia among these pregnant women and its associated risk factors with the aim of preventing short- and long-term adverse effects to the mother and her newborn by linking her to specialized care if found with hyperglycaemia.

## 2. Materials and Methods

### 2.1. Study Design and Site

Our study followed a cross-sectional design. We conducted it at Itojo General Hospital, a government health facility which is found in the south western part of Uganda located in Ntungamo District, approximately 340 km from Kampala city. This hospital offers both in-patient and outpatient services at various clinics housed within the facility and also has an estimated bed capacity of 120. We carried out study at the antenatal clinic of this hospital. The clinic receives approximately 230 pregnant women per month of which most of them are on their first visit. This clinic operates 5 days a week and receives an average of 12 women on a daily basis. In this hospital, women with noncomplicated conditions receive outpatient treatment from the antenatal clinic, whereas those with maternal danger signs are transferred to the maternity ward for further management. Although glucose testing has been widely recognized as a mandatory test during pregnancy, this is not true for the pregnant women receiving antenatal care at the Itojo General Hospital mainly due to the limited funding the hospital receives from the Ministry of Health and from the government of Uganda. The hospital therefore allocates funds to a specific number of baseline tests such as HIV/AIDS testing as a mandatory free test, and the rest are done at a fee.

### 2.2. Study Flow

We collected research data from pregnant women at this facility for a period 3 months (October to December 2019). In this cross-sectional study, we aimed to include consenting pregnant women without a known history of diabetes mellitus and were receiving antenatal care at the study site. We excluded potential participants who had a known personal history of diabetes mellitus. While recruiting participants, we followed the systematic sampling method whereby we selecting every second pregnant woman who met the inclusion criteria. The sample size of the study population was determined using the formula by Kish and Leslie (1965) for cross-sectional studies [11]:

(1) $$\begin{array}{c} n=Z^{2}P\;\left(1–P\right)/W^{2} \end{array}$$

where *n* is the minimum sample size, *Z* = 1.96 (for 95% confidence interval), *P* is the estimated frequency of hyperglycaemia first detected in pregnancy to be 31.9% [6], and *W* is the margin of error to be 5%. Thus, 323 participants were recruited into the study.

**Figure 1:**
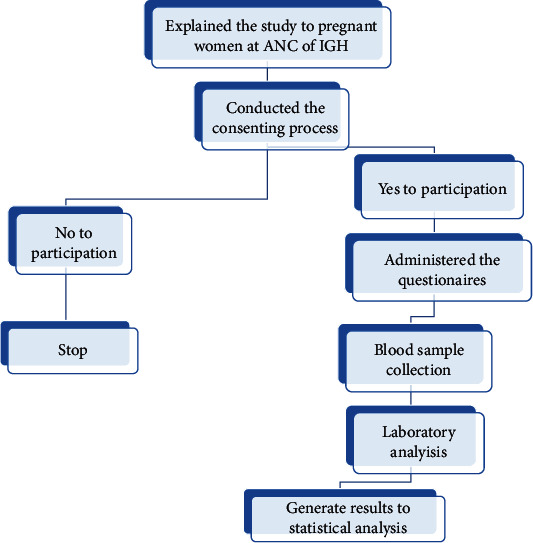
The flow of the study showing the sequence of the activities in the study.

The sequence of the activities on how participants were enrolled into the study is summarised in Figure 1 below. We commenced with explaining the purpose and procedures involved in the study followed by obtaining consent of the eligible participants. The consenting participants were subjected to a fine-tuned questionnaire with close-ended questions used to collect the study participants' social demographic information and economic statuses. Data on the clinical history of the participant such as that on gravidity, number of live births, abortion or miscarriage information, and the presence of a family history of diabetes mellitus were obtained. Anthropometric measurements such as determination of the systolic and diastolic pressure were measured on the day of enrolment into the study using a mercury sphygmomanometer with small (<21 cm) and normal (22-32 cm) cuff sizes on the left arm at the level of the heart while the patient was seated. Weight in kilograms was determined using a weighing scale machine making sure that the she had no heavy clothing or shoes; height was measured to the nearest 0.1 cm against a vertical wall. The study participant's weight in kilograms and the square of their height in meters were used to calculate their body mass index (BMI). Biochemical assessment on the participants' glucose levels was done to ascertain hyperglycaemia in pregnancy.

Data was collected by determining the blood glucose levels of the study participants who came for antenatal services. Only potential participants, who met the criteria and had consented to participate in the study while following the systematic random sampling method. In this case, the principle investigators explained the study details to the potential participants and conducted the consenting process. Informed written consent was obtained from those who agreed to participation into the study.

### 2.3. Diagnosis of Gestational Hyperglycaemia

At the first encounter with the study participant, random blood sugar (RBS) test samples were collected by capillary blood collection and tested using a glucometer (one touch select), and those who had random glucose levels between 140.4 mg/dL (7.8 mmol/L) and 198 mg/dL (11.0 mmol/L) did not undergo any further glucose testing while those who had higher than 200 mg/dL (11.1 mmol/L) were subjected to further testing. In order to conduct a fasting blood sugar (FBS) test, the study participants who turned out to have abnormal RBS levels were encouraged to continue with their normal food diet for 3 days and return to the testing centre for further blood sampling. On the night before returning, they undertook an overnight fast of approximately 8 to 12 hours and came to the study site the following morning for subsequent testing. Fasting venous blood samples were obtained from each participant's upper right arm following venous blood collection protocols, and the 4 mL of blood collected was placed into fluoride oxalate coated specimen bottles to minimise glycolysis. The glucose concentrations in this study were determined using a colorimeter *WPA Cambridge, model: C07000 Company: Roche* with a glucose reagent kit that used the principle of glucose oxidase (GOD) method, manufactured by *Labtest Diagnostica*, and it was used following the manufacturer's instructions encrypted on the test kit's working manual. Standard glucose solutions were used to monitor the testing procedures as a form of quality control. According to our study, the fasting blood sugar test is what we used to identify the cases of hyperglycaemia in pregnancy. If the values were equal to or greater than 95.5 mg/dL (5.3 mmol/L), the participant was noted as a hyperglycaemic case with no further blood work being done. A questionnaire was administered to the study participant to collect data on other precipitating factors of the condition. The glucose tests in this study were performed by well-trained laboratory technicians at the Itojo General Hospital. The criteria for the interpretation of test results on hyperglycaemia in pregnancy includes a random plasma sugar of ≥11.1 mmol/L (200 mg/dL) in the presence of diabetic symptoms and/or a fasting plasma glucose of 5.1–6.9 mmol/L (92–125 mg/dL) [12].

This study obtained clearance from the department of medical Laboratory Science and ethical clearance from the Faculty of Medicine Research and Ethics Committee (FREC) at Mbarara University of Science and Technology. During recruitment into the study, the participants were required to provide informed written consent by way of signing the consent document, and those who could not read or write were requested to place their thumb print on the signature blank. By doing so, they were agreeing to participate in the study. The participants were fully oriented to the fact that they would meet their transportation costs to the facility especially in the case of those who would be required to undertake a second blood glucose test such as in fasting blood sugar measurements. The participants had the benefit of knowing their test results from the random blood sugar and fasting blood sugar analysis. Those who turned out to have hyperglycaemia were linked to the specialists while some were admitted to the maternity ward within the hospital for further management. Those patients, who did not wish to participate in the study, did not have any disturbances in their normal prenatal care, and those who had been enrolled had the right to leave the study at any point they wished to opt out.

### 2.4. Data Analysis

Data collected were analyzed using the Statistical Package for Social Science™ (SPSS) Version 26. Descriptive statistics, such as maternal age, level of education, and occupation, were analyzed as frequencies, mean, and percentages. Bivariate and multivariate analysis was carried out to reveal any statistically significant differences among the maternal risk factors (age, gravidity, and level of education, body mass index (BMI), occupation, number of live children, history of abortion or miscarriage, awareness of gestational hyperglycaemia, a family history of diabetes mellitus, and trimester and blood pressure levels) and the presence of hyperglycaemia in pregnancy. Differences between proportions were assessed by Fisher's exact test. Odds ratios and *P* values were used to interpret these relationships. The outputs were presented as tables and figures for easy comprehension. In our study, a *P* value < 0.05 was considered statistically significant.

## 3. Results

The enrolled sample size of this study was 323 participants. Of these, only 307 participated in the study to completion. The 16 participants withdrew their consent at the point of blood collection, and this gave our study a 95.04% response rate.

A total of 307 pregnant women enrolled into the study were able to participate until completion. Of these, 48 (15.64%) were diagnosed with hyperglycaemia in pregnancy. The mean age of study participants was 26.42 (±5.7) (see Table 1).

**Figure 2:**
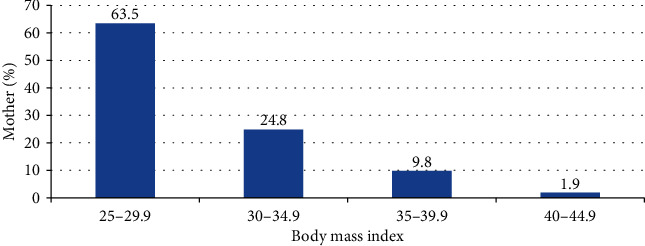
Bar graph showing the percentage of mothers against their body mass index.

**Figure 3:**
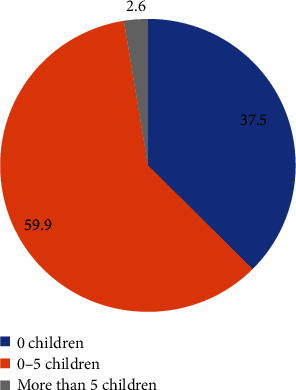
Percentage of mothers with live children.

**Figure 4:**
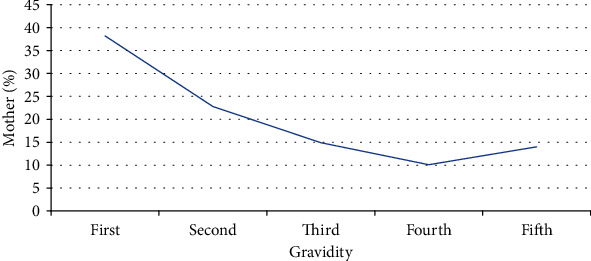
Line graph showing the percentage of mothers against their gravidity.

The descriptive characteristics established in this study included the assessment of body mass index (BMI), number of live children, and gravidity of participants to determine their association to gestational hyperglycaemia. Only 6 (1.9%) of the respondents were obese, majority (195 (63.5%)) of the respondents had their BMI 25 to 29.9, and the mean BMI is 33.6. Also to note is that majority of the respondents (184 (59.9%)) had an average of 1 to 5 live children with only 8 (2.6%) of the respondents having over 5 live children. Considering gravidity of the participants, most of them were prime gravid mothers, 117 (38.9%). Those who were carrying more than the fifth pregnancy were 40 (0.9%). The characteristics of the study population are summarised as depicted in Figures 2–4.

Bivariate analysis of gestational hyperglycaemia, maternal age, and parity of study participants is summarised in Table 2.

Multivariate analysis of maternal risk factors associated with gestational hyperglycaemia is summarised in Table 3.

## 4. Discussion

Our study revealed a frequency of hyperglycaemia first detected in pregnancy among study participants to be at 15.6%. This result was both in agreement and disagreement with some studies conducted in other African countries. In a study conducted in Cameroon, the frequency of hyperglycaemia among pregnant women was 20.5% [13]. This study employed similar diagnostic methods in identification of hyperglycaemic cases as used in our current study. However, we believe that the difference in the frequency could have been due to the different sizes of the study population. A systematic review conducted on studies done in African countries on hyperglycaemia in pregnancy indicated four South African studies to have a frequency ranging from 1.6% to 8.8% [14]. The issue about all four of these studies is the fact they all utilized a 2-hour 50 g OGTT and the investigators' own diagnostic criteria when identifying their cases, and this is different from what was done in our current study, thus the observed low frequency in their studies. The procedure of screening with 50 g Oral Glucose Tolerance Test (OGTT) was not feasible in a rural setting like our current study site because the pregnant women had to be strictly monitored and would have been required to visit the antenatal care clinic twice, and at least three to five blood samples would have been drawn. This fact alone would likely reduce patients' compliance to follow-up. Contrary to our findings is the frequency of hyperglycaemia in pregnant women obtained from two studies conducted in Tanzania and Rwanda, which was found to be 5.6% (as the overall frequency, 8.9% was the contribution from the urban and 1.0% from the rural areas) [15] and 3.2% [16], respectively. Likewise, these two studies also used the similar diagnostic criteria as those conducted in South Africa. In general, the frequency rate observed in this study was within the universal range varying from 8% to 20.7% among all pregnancies in different populations across the African continent and the global range of 3 to 16% across the world [14]. More on the frequency are the disagreeing findings from studies conducted in Rwanda (8.3%) [17], Tanzania (5.6%) [15], Egypt (8%) [18], and Nigeria (8.6%) [19] which obtained a lower frequency than what was observed in our study and that which was reported by the Management Sciences for Health [20] who conducted a study of hyperglycaemia in pregnancy among urban women in Tigray, Northern Ethiopia, and obtained a frequency of 15.8%. The main reason for the high frequency of gestational hyperglycaemia in this study setting might have been due to the fact that the lower cut-off points for fasting plasma glucose (FPG) were used as required in the updated diagnostic criteria. On the other hand, the findings from studies conducted in Tanzania (19.5%) [21] and South Africa (25.8%) [22] which used a similar diagnostic criteria revealed a much higher frequency. This evidence indicates that lack of a gold standard for the actual diagnosis of hyperglycaemia in pregnancy is responsible for observed heterogeneity in the study's results, and this might also be affected not only by the different diagnostic criteria but also by the characteristics of the population [23]. Increased awareness for hyperglycaemia in pregnancy, change in lifestyle, and the rising frequency of obesity might have contributed the varying outcomes in the frequency status. A review study, which examined the factors associated with hyperglycaemia in pregnancy at Mulago National Referral Hospital in Uganda, reported that the variations in the frequency rates of the diagnosed hyperglycaemia in pregnancy could be attributed to multiple factors, such as the use of different criteria for diagnosis, increases in the rates of obesity, and limitation of the conducted surveys [23].

Although not all the potential risk factors showed independent and/or significant associations with hyperglycaemia first detected in pregnancy, the effect of these risks could be influenced by moderating or confounding variables. Our study established a significant correlation between high body mass index (BMI) and the risk of having hyperglycaemia in pregnancy. The participants who had a body mass index above 40 also had abnormal values of fasting plasma glucose and were found to be 40% of the study population. Different studies in Africa such as [19] assessed obesity using BMI and found a significant association with hyperglycaemia in pregnancy and thus confirming our findings. Similarly, a review and meta-analysis by [24] revealed that prepregnancy BMI was more strongly associated with the risk of hyperglycaemia in pregnancy. This was due to the fact that the decreased insulin sensitivity in obese pregnancies increased the blood glucose level [24]. This implies that an overweight individual who is exposed to a sedentary lifestyle is likely to become obese, and this cycle also adversely affects the glucose metabolism.

Significant risk factors for hyperglycaemia in pregnancy in our study such as a history of a previous abortion and prime parity are in agreement with a study which was done at the Mulago National Referral Hospital, Uganda, where 56.7% of cases with hyperglycaemia in pregnancy were of low parity [25] and an early maternal age (19-30 years). The conformity in these studies could be explained by the fact that both were conducted in the same country meaning that the populations in the study are under the influence of similar government and public health policies which affect citizens in a somewhat similar manner. For example, the early marriages imply early maternal age, and this is a serious a public health concern for the government of Uganda. However, our study conflicts with a study done in Australia which showed that hyperglycaemia in pregnancy increases with advanced age [26], being in the second trimester of pregnancy and having one or more live children. Apart from the second trimester of pregnancy, the rest of our findings on risk factors for hyperglycaemia in pregnancy were consistent with other studies [27]. A study has attributed the occurrence of hyperglycaemia in pregnancy to increases in body mass index as a high maternal weight is associated with a substantially higher risk of gestational diabetes mellitus [28]. It is noteworthy that the mean BMI in our study was significantly high, with 36.5% participants being overweight which confirms that increased BMI is a risk factor for hyperglycaemia in pregnancy despite the young age of mothers and their parity status. Although the calorific value of their nutritional intake was not ascertained, the social valorization of stoutness was believed to be the predisposing factor to obesity among mothers in Itojo and Uganda in general [28]. Some studies have related the presence of high glucose levels in pregnancy to the higher frequency of stillbirths. A multicentre, randomized, controlled trial that was conducted on 440 pregnant women (220 with hyperglycaemia in pregnancy and 220 controls) in Saudi Arabia found that stillbirth during previous pregnancy was not a significant predictor for hyperglycaemia in pregnancy. Our study could not comment on this outcome as it was not assessed. However, this is recommended for further studies as the cause of stillbirths is a rarely investigated factor, let alone the inappropriate documentation and education of the same to these mothers on the cause of the birth outcomes who are still wanting. Our findings also indicated peasantry as a significant risk factor to hyperglycaemia in pregnancy. This finding emphasizes that the relationship between peasantry dietary diversity cannot be overlooked as it somewhat plays a role in the control of glycaemic levels varied across a range of factors related to the socioeconomic status of individuals and households. In our study, most of the participants were peasants that would find it difficult to rationalize nutritious metabolic dietary options considering the cost of living in the semiurban Ntungamo District in South Western Uganda versus their household income.

Our study had some limitations. Firstly, we conducted a hospital-based cross-sectional study instead of a follow-up community-based study thus making inference to the general population difficult. However, the hospital where the study was conducted has a large catchment area with patients from many rural villages surrounding the area, and the findings here generally reflect the picture in the study area. Secondly, we could not study other relevant risk factors as cited by literature such as polycystic ovarian syndrome (PCOS), gestational weeks, maternal eating habits, and stress among others because they needed additional time and costs, yet this was a defunded study. Then, we used body mass index (BMI) of the patients at the time of the study instead of prepregnancy BMI which is the standard. It should be noted that prepregnancy records for demographics like BMI are rarely kept in the rural areas of Uganda.

## 5. Conclusions

This study has indicated new information about the frequency and risk factors of hyperglycaemia in a rural community of South Western Uganda. The frequency of hyperglycaemia during pregnancy at Itojo General Hospital is 15.6% and prime parity; maternal age of 19-30 years is the potential risk factor for the outcome. The study also found that a history of a previous stillbirth among other factors as insignificant risk factors for gestational hyperglycaemia. The results of this study therefore provide preliminary information which is relevant in the decision making on health programs such as those advocating for early screening, diagnosis, and treatment of hyperglycaemia in pregnancy which is also a serious maternal health concern.

## Figures and Tables

**Table 1:** Sociodemographic characteristics, *N* = 307.

Variable	Frequency	Percentage
Age		
15-18	16	5.2
19-24	125	40.8
25-30	110	35.8
31-36	28	9.1
37-42	28	9.1
Level of education		
None	3	0.9
Primary	193	63.4
Secondary	78	25.0
Tertiary	33	10.7
Occupation		
Business	110	35.8
Civil servant	16	5.2
None	9	2.9
Peasant	169	55.2
Student	3	0.9

**Table 2:** Gestational hyperglycaemia by maternal age and parity of study participants, *N* = 307.

Factor	Gestational hyperglycaemia		Odds ratio	95% CI	*P* value
	Normal, *n* (%)	Abnormal, *n* (%)			
Age					
15-18	10 (62.5)	6 (37.5)	1		
19-24	116 (92.8)	9 (7.2)	7.73	2.29-26.15	0.001∗
25-30	95 (86.4)	15 (13.6)	3.8	1.20-11.99	0.023∗
31-36	22 (78.6)	6 (21.4)	2.2	0.57-8.53	0.255
37-42	15 (55.6)	12 (44.4)	0.75	0.21-2.66	0.656
Parity					
First	111 (94.9)	6 (5.1)	5.37	1.78-16.25	0.003∗
Second	56 (80.0)	14 (20.0)	1.16	0.45-3.00	0.757
Third	35 (76.1)	11 (23.9)	0.923	0.34-2.52	0.877
Fourth	25 (80.7)	6 (19.4)	1.201	0.38-3.87	0.748
Fifth	31 (77.5)	9 (22.5)	1		

∗Significant at *P* < 0.05.

**Table 3:** Maternal risk factors associated with gestational hyperglycaemia, *N* = 307.

Factor	Gestational diabetes mellitus		Odds ratio	95% CI	*P* value
	Normal, *n* (%)	Abnormal, *n* (%)			
Level of education					
None	2 (100.0)	0 (0.0)	1		
Primary	158 (81.9)	35 (18.3)	0.62	0.21-1.88	0.402
Secondary	69 (88.5)	9 (11.5)	1.057	0.30-3.71	0.930
Tertiary	29 (87.9)	4 (1 2.1)	1		
Body mass index					
30-34.9	202 (83.8)	39 (16.2)	1		
35-37.9	53 (88.3)	7 (11.7)	1.461	0.62-3.45	0.387
Above 40	3 (60.0)	2 (40.0)	0.289	0.047-1.79	0.182
Occupation					
Business	106 (96.4)	4 (3.6)	1		
Civil servant	14 (87.5)	2 (12.5)	0.264	0.044-1.576	0.144
None	9 (100.0)	0 (0.0)	1		
Peasant	129 (76.3)	40 (23.7)	0.121	0.042-0.35	0.001∗
Student	0 (0.0)	2 (100.0)	1		
Number of live children					
0	109 (94.8)	6 (5.2)	1		
1-5	147 (79.9)	37 (20.1)	0.218	0.09-0.5365	0.001∗
Above 5	2 (28.6)	5 (71.4)	0.02	0.004-0.138	0.001∗
Abortion					
No	247 (86.4)	39 (13.6)	1		
Yes	11 (55.0)	9 (45.0)	0.192	0.075-0.496	0.001∗
Awareness of gestational hyperglycaemia					
No	195 (84.1)	37 (15.9)	1		
Yes	63 (85.1)	11 (14.9)	1.086	0.52-2.256	0.823
Family history of hyperglycemia					
No	231 (85.2)	40 (14.8)	1		
Yes	27 (77.1)	8 (22.9)	0.58	0.25-1.38	0.219
Trimester					
1st	34 (100.0)	0 (0.0)	1		
2nd	36 (70.6)	15 (29.4)	0.42	0.21-0.85	0.017∗
3rd	188(85.1)	33(12.9)	1		
Diastolic blood pressure					
40-59	108 (85.7)	18 (14.3)	1		
60-79	32 (84.2)	6 (15.8)	0.889	0.33-2.43	0.818
80-99	118 (83.1)	24 (16.9)	0.819	0.42-1.59	0.557
Systolic blood pressure					
120-139	38 (80.9)	9 (19.2)	1		
70-89	17 (89.5)	2 (10.5)	2.01	0.39-10.33	0.402
90-119	203 (84.6)	37 (15.4)	1.299	0.58-2.91	0.524

∗Significant at *P* < 0.05.

## Data Availability

Data is available upon request from the corresponding author.
